# Serum Biomarkers and Their Association with Myocardial Function and Exercise Capacity in Cardiac Transthyretin Amyloidosis

**DOI:** 10.3390/jcdd11050142

**Published:** 2024-04-30

**Authors:** Luis Nieto-Roca, Andrea Camblor Blasco, Ana Devesa, Sandra Gómez-Talavera, Jorge Balaguer-Germán, Jairo Lumpuy-Castillo, Ana María Pello, Luis Martínez Dhier, Gregoria Lapeña, Lucía Llanos Jiménez, Óscar Lorenzo, José Tuñón, Borja Ibáñez, Álvaro Aceña

**Affiliations:** 1 Department of Cardiology, IIS-Fundación Jiménez Díaz University Hospital-Quironsalud, Avenida Reyes Católicos, 2, 28040 Madrid, Spain; luisnietor93@gmail.com (L.N.-R.); acamblor@hotmail.com (A.C.B.); sandra.gomez@externo.cnic.es (S.G.-T.); jorge.balaguer@quironsalud.es (J.B.-G.); ampello@quironsalud.es (A.M.P.); jtunon@quironsalud.es (J.T.); bibanez@fjd.es (B.I.); 2 Centro Nacional de Investigaciones Cardiovasculares Carlos III (CNIC), Melchor Fernández Almagro, 3, 28029 Madrid, Spain; adevesaarbiol@gmail.com; 3 Zena and Michael A. Weiner Cardiovascular Institute, Icahn School of Medicine at Mount Sinai, New York, NY 10029, USA; 4 Centro de Investigación Biomédica en Red de Enfermedades Cardiovasculares (CIBERCV), 28029 Madrid, Spain; 5 Laboratory of Diabetes and Vascular Pathology, IIS-Fundación Jiménez Díaz, Medicine Department, Universidad Autónoma, 28040 Madrid, Spain; jlumpuy@gmail.com (J.L.-C.); olorenzo@fjd.es (Ó.L.); 6 Spanish Biomedical Research Centre on Diabetes and Associated Metabolic Disorders (CIBERDEM) Network, 28040 Madrid, Spain; 7 Department of Nuclear Medicine, IIS-Fundación Jiménez Díaz University Hospital-Quironsalud, Av. Reyes Católicos, 2, 28040 Madrid, Spain; lmartinez@quironsalud.es (L.M.D.); glapena@fjd.es (G.L.); 8 Research Unit, IIS-Fundación Jiménez Díaz, 28002 Madrid, Spain; lucia.llanos@quironsalud.es; 9 Faculty of Medicine, Universidad Autónoma de Madrid, 28029 Madrid, Spain

**Keywords:** cardiac amyloidosis, transthyretin amyloidosis, atrial strain, ventricular strain, inflammation, MCP-1, IL-6, hsCRP, klotho

## Abstract

Background: Transthyretin cardiac amyloidosis (ATTR amyloidosis) is a frequent etiology of heart failure. Inflammation and mineral metabolism are associated with myocardial dysfunction and clinical performance. Cardiac global longitudinal strain (GLS) allows function assessment and is associated with prognosis. Our aim was to describe possible correlations between GLS, biomarker levels and clinical performance in ATTR amyloidosis. Methods: Thirteen patients with ATTR amyloidosis were included. Clinical characteristics; echocardiographic features, including strain assessment and 6 min walk test (6MWT); and baseline inflammatory, mineral metabolism and cardiovascular biomarker levels were assessed. Results: Of the 13 patients, 46.2% were women, and the mean age was 79 years. TAPSE correlated with NT-ProBNP (r −0.65, *p* < 0.05) and galectin-3 (r 0.76, *p* < 0.05); E/E′ ratio correlated with hsCRP (r 0.58, *p* < 0.05). Left ventricular GLS was associated with NT-ProBNP (r 0.61, *p* < 0.05) (patients have a better prognosis if the strain value is more negative) and left atrial GLS with NT-ProBNP (r −0.73, *p* < 0.05) and MCP1 (r 0.55, *p* < 0.05). Right ventricular GLS was correlated with hsTnI (r 0.62, *p* < 0.05) and IL6 (r 0.881, *p* < 0.05). Klotho levels were correlated with 6MWT (r 0.57, *p* < 0.05). Conclusions: While inflammatory biomarkers were correlated with cardiac function, klotho levels were associated with clinical performance in the population with TTR-CA.

## 1. Introduction

Heart failure with preserved ejection fraction (HFpEF) is a very common condition with an increasing prevalence, due to the progressive aging of the population. In addition, HFpEF is a frequent manifestation of several underlying myocardial diseases with different prognoses, clinical manifestations and management. Therefore, there is a pressing need to identify the etiology when facing a patient with HFpEF.

Cardiac amyloidosis is a frequent etiology of HFpEF. Recent evidence has shown that transthyretin cardiac amyloidosis (ATTR amyloidosis) is one of the most prevalent forms, ranging from 6 to 13% in large-cohort registries of patients with HFpEF [1,2,3]. ATTR amyloidosis is the result of a misfolding of a carrier protein mainly synthesized in the liver, leading to its deposition as amyloid material in different organs, especially in the myocardium [4]. ATTR amyloidosis comprises ATTRv (v for variant) amyloidosis and ATTRwt (wt for wild type) amyloidosis; the latter is the most frequent. Due to this genetic component, early diagnosis is essential, implying possible clinical repercussions, especially in terms of prognosis, management and genetic counselling (not just for the patients but also for their relatives).

It is important to point out that clinical suspicion of amyloid deposits in HFpEF patients is usually low, as they may have alternative explanations for left ventricular wall (LV) thickening and diastolic dysfunction (chronic hypertension, chronic kidney disease, etc.). Echocardiographic features are also variable, can be misleading and, in the majority of cases, are non-specific. Nevertheless, when several findings are present such as “cherry-on-top” apical sparing strain, increased left and right ventricular (RV) thickness, atrial enlargement, restrictive LV filling pattern and pericardial effusion, a “red flag” can be raised and a diagnostic work-up searching for ATTR amyloidosis should be initiated. In addition, ATTR amyloidosis is concomitantly present in a high percentage of patients [5]. Some of these features have also been associated with disease progression and prognosis in ATTR amyloidosis [6].

Fortunately, the development of non-invasive diagnostic techniques such as technetium-labelled cardiac scintigraphy has allowed feasible non-invasive diagnosis with high specificity, especially for ATTR amyloidosis [7].

On the other hand, biomarkers are rapidly spreading in the HFpEF field, not just for disease diagnosis but also for risk stratification and prognosis. Cardiac amyloidosis is no exception and recently the focus has moved to biomarker-based prognostic scores and staging systems for AL and ATTR amyloidosis. These biomarkers usually refer to myocardial involvement (such as troponin T and natriuretic peptides) and renal function (estimated glomerular fraction rate (eGFR)). Other types of biomarkers have been proposed, especially for the early and mid-stages of the disease, and are mainly related to systemic inflammation and amyloid formation [8]. 

Despite its prevalence and clinical relevance, there are very few clinical trials focused on ATTR amyloidosis [9,10,11] and the vast majority of evidence comes from observational studies. In addition, there are real gaps in evidence regarding the use of echocardiographic parameters and biomarkers as tools for predicting prognosis and clinical results in patients with ATTR amyloidosis.

The aim of our study is to describe the possible correlations between echocardiographic characteristics and clinical performance with certain biomarker levels.

## 2. Methods

### 2.1. Patients and Study Design

The basal characteristics of 13 patients with a diagnosis of ATTR amyloidosis were analyzed. These patients were part of a prospective clinical trial (EUDRACT number 2019-002873-80) to assess if they were candidates for diflunisal treatment at the time of diagnosis, being included from March 2021 to May 2022. Diagnosis was made according to the latest ESC position statement on the diagnosis and management of cardiac amyloidosis non-invasive criteria [12] (suggestive echocardiographic or CMR parameters, grade 2 or 3 cardiac uptake at diphosphonate scintigraphy and negative serum-free light chains and negative serum and urine immunofixation), and patients were included before any amyloid drugs (tafamidis, patisiran or diflunisal) were initiated.

This study was approved by the institutional ethics committee (EUDRACT number 2019-002873-80 and PIC54-2017). 

Data including baseline clinical characteristics, cardiovascular risk factors, comorbidities, GFR calculated by Chronic Kidney Disease Epidemiology Collaboration (CKD-EPI) equation, presence of amyloid-compatible clinical signs (carpal tunnel syndrome, lumbar stenosis, polyneuropathy, tendinopathy and dementia), electrocardiographic (rhythm, heart rate and QRS complex width) and echocardiographic findings, Perugini grading according to cardiac scintigraphy, New York Heart Association (NYHA) functional class and type and dose of cardiovascular drugs at the start of follow-up were collected. 

Additional tests were also performed: a complete baseline 2D echocardiogram [including LV, RV and left atrial (LA) strain studies]; a 6-min walk test (6MWT); and blood tests including baseline levels of cardiovascular, inflammatory [NT-proBNP, NT-ProANP, high-sensitivity Troponin I (hsTnI), creatine kinase-myocardial band (CK-Mb), high-sensitivity C-reactive protein (hsCRP), galectin 3 (GLT3), monocyte chemoattractant protein 1 (MCP1), interleukin 6 (IL6) and T cell immunoglobulin and mucin domain 1 (TIM1)] and mineral metabolism biomarkers [parathormone (PTH), calcidiol (25OH-vitD), fibroblast growth factor 23 (FGF23) and klotho].

### 2.2. Echocardiography

Standard transthoracic echocardiography was performed by a physician with expertise in cardiac imaging using a commercially available system (EPIQ 7C, Philips Medical Systems, Andover, MA, USA). Echocardiography data were stored digitally. Ultrasound studies were later analyzed with QLab9 (Philips Healthcare, Bothel, WA, USA) at the Centro Nacional de Investigaciones Cardiovasculares (CNIC) Core Imaging Laboratory. Imaging quality was evaluated as optimal, suboptimal or non-interpretable, and the inclusion of studies was determined by consensus. Ejection fraction (EF) and left atrial volume (LAV) were measured with the biplane Simpson method in apical 4- and 2-chamber views, with LAV indexed over body surface area. LV mass and mass index (LVMI) were assessed through the Cube formula. LV wall thickness was assessed in a long parasternal axis using 2D techniques. LV hypertrophy was defined as an interventricular septum thickness > 12 mm. Myocardial volume was calculated as the ratio of LV mass over 1.05 (myocardial density). The mitral inflow early (E) and late diastolic (A) velocities, the (E/A) ratio, the deceleration time of the E wave (DT) as well as the pulsed-wave tissue Doppler early diastolic mitral velocity (E′) of the medial and lateral annulus were measured. Right ventricular size was defined using basal end-diastolic diameter; function was defined by assessing the tricuspid annular plane systolic excursion (TAPSE) and the S wave acquired by the pulse-wave tissue Doppler. Doppler tracings of the aortic and mitral valves were used to define end-systole and end-diastole. For patients in sinus rhythm all reported measurements were averaged over 3 cardiac cycles; atrial fibrillation (AF) measurements were averaged over 5 cycles.

LV, RV and LA global longitudinal strain (GLS) measurements were performed offline as recommended. Digitally acquired clips were considered suitable for offline 2D Speckle Strain Imaging analysis if at least three cardiac cycles were available, with high frame rates (70 to 100 frame/s) and without dropout of more than one LV, RV or LA segment. The endocardial border was traced at the end-diastolic frame in the apical view. End-diastole was defined by the QRS complex or by the frame just before mitral valve closure. The software tracked speckles along the endocardial and epicardial borders throughout the cardiac cycle, and the width of the region of interest was adjusted to fit the entire myocardium. All strain variables are the result of the mean measures using 3 cardiac cycles regardless of baseline rhythm. LV strain and all derived variables were measured in apical 4-, 2- and 3-chamber views. LA strain data refer to the LA strain reservoir, measured at the end of the reservoir phase as the average of the peak systolic strain from 12 segments in 3 cardiac cycles.

### 2.3. Six-Minute Walk Test

Regarding the 6MWT, in a quiet 40 m hall, the patients were instructed to walk as fast and perform as many laps as possible between the distance markers over a period of 6 min. The walk test was supervised but unencouraged. Blood pressure and heart rate were measured at the start and at the end of the test. Patients were allowed to stop and rest if needed. The total distance walked was recorded.

### 2.4. Biomarkers Determination

For inflammatory biomarker determination, twelve-hour fasting venous blood samples were withdrawn and collected in EDTA. Blood samples were centrifuged at 2500× *g* 13 for 10 min and plasma was stored at −80 °C. Plasma concentrations of human MCP1, hsCPR, TIM 1, IL-6 and galectin 3 were measured using the automated immunoassay system ELLA from Protein Simple (Bio-Techne, Minnesota, USA) according to the manufacturer’s instructions. The detection kits were SPCKA-PS-008754 (TIM1 and IL-6), SPCKB-PS001108 (MCP1), SPCKB-PS000200 (hsCRP) and SPCKB-PS000490 (galectin-3). Each plasma sample was analyzed in triplicate, and the inter-plate coefficient of variation was less than 4% in all cases. The levels of plasma human NT-ProANP were assessed by an immunoassay using a specific kit (ref: DANP00) from R&D Systems; high-sensitive troponin (Hs-TnI) levels were assessed by direct chemiluminescence (ADVIA Centaur; Siemens, Berlin, Germany); and those of human creatine kinase-myocardial band (CK-Mb) were achieved by an immunoassay using VITROS Immunodiagnostic products (ref: 1896836) at the Analytical Service of the Fundación Jiménez Díaz.

Regarding the mineral metabolism biomarkers, plasma calcidiol levels were quantified by a chemiluminescent immunoassay (CLIA) on the LIAISON XL analyzer (LIAISON 25OH-Vitamin D total Assay Dia Sorin, Saluggia, Italy); FGF23 was measured by an enzyme-linked immunosorbent assay which recognizes epitopes within the carboxyl-terminal portion of FGF-23 (Human FGF23, C-Term, Immutopics Inc., San Clemente, CA, USA); klotho levels by ELISA (Human soluble alpha klotho assay kit, Immuno-Biological Laboratories Co., Gunma, Japan); and intact parathormone was analyzed by a second-generation automated chemiluminescent method (Elecsys 2010 platform, Roche Diagnostics, Mannheim, Germany).

This investigation was carried out in accordance with the principles outlined in the Declaration of Helsinki. 

### 2.5. Statistical Analysis

Data were subjected to descriptive statistical analysis via frequency measurements (absolute frequencies and percentages) for qualitative variables and displayed as median (interquartile range) for quantitative variables. 

Kolmogorov–Smirnov or Shapiro–Wilk tests were used to determine normal or non-normal distribution for each variable.

In order to establish the presence or absence of an association between different quantitative variables for the 13 patients, we calculated either the Pearson or Spearman Rho correlation coefficient (r). 

Analyses were performed with SPSS 19.0 (SPSS Inc., New York, NY, USA). Statistical differences were considered significant at *p* < 0.05 (two-tailed).

## 3. Results

### 3.1. Baseline Characteristics

During the study period, 13 patients with ATTR amyloidosis were assessed for eligibility. Table 1 shows the baseline characteristics of the total population. In total, 46.2% were women, and the mean age was 79 years. Regarding cardiovascular risk factors, 69.2% were hypertensive; 30.8% were diabetic; and 76.9% were dyslipidemic. Overall, 57.1% had chronic kidney disease (eGFR < 60 mL/m/m^2^), 69.2% of the subjects were at sinus rhythm at inclusion, and low voltage was described in 23.1%. The median LVEF was 52%. Median NT-ProBNP levels at inclusion were 1340 pg/mL. A total of 52% of the patients did manifest extracardiac clinical signs of ATTR amyloidosis (carpal tunnel syndrome, lumbar spinal stenosis, polyneuropathy or bicep tendon rupture).

Regarding medical treatment, seven patients (53.8%) were receiving angiotensin-converting enzyme inhibitor (ACEIs) or angiotensin receptor blockers (ARBs) and six (46.2%) were taking BB at the beginning of the follow up. Furthermore, 61.5% of the cohort was taking loop diuretics.

### 3.2. Baseline Features of the Subgroup Analysis

Table 2 shows the results for baseline echocardiographic features, biomarker levels and the 6MWT for the 13 patients. The strain data showed significant absolute low values. For LV GLS, the median was −10% [IQR (−15–−6)]; for RV GLS, the median value was −10.3 [IQR (−11.5–−6.1)]; and for LA GLS, the median was 9.2 [IQR (3.5–11.0)]. 

### 3.3. Correlations between Biomarkers and Myocardial Disfunction

We analyzed the possible correlations between myocardial function, including strain values, biomarkers and the distance in the 6MWT. These results are shown in Table 3.

No correlations were found between LVEF and any kind of biomarker included. TAPSE correlated with NT-ProBNP levels (r −0.65, *p* < 0.05) and galectin-3 (r 0.76, *p* < 0.05). The E/E′ ratio had a direct correlation with hsCRP (r 0.58, *p* < 0.05).

Regarding the strain values, LV GLS correlated with NT-ProBNP levels (r 0.61, *p* < 0.05) (more negative strain values are associated with better myocardial performance and better prognosis) (r −0.73, *p* < 0.05). In addition, LA GLS was significantly associated with MCP1 levels (r 0.55, *p* < 0.05). RV GLS was significantly associated with hsTnI (r 0.62, *p* < 0.05) and IL6 (r 0.881, *p* < 0.05) levels.

The klotho baseline levels were significantly correlated with the 6MWT results (r 0.57, *p* < 0.05).

We also analyzed the different correlations regarding LVEF. Taking into account that all patients had an LVEF > 40%, we selected an LVEF of 60% as our threshold. In those patients with an LVEF < 60%, NT-ProBNP levels were significantly correlated with LA GLS (r 0.55, *p* < 0.05); IL6 levels were inversely associated with the 6MWT results (r −0.94, *p* < 0.05) and directly with hsCPR levels (r 0.96, *p* < 0.05); galectin-3 was associated with hsTnI (r 0.90, *p* < 0.05); and CKMB was inversely associated with LV GLS (r −0.75, *p* < 0.05). On the other hand, in patients with LVEF > 60%, NT-ProBNP levels did inversely correlate with LA GLS (r −0.71, *p* < 0.05), whereas IL6 was directly associated with hsTnI (r 0.99, *p* < 0.05). The klotho baseline levels did not have any significant correlations in either subgroup of patients.

## 4. Discussion

The major findings in this ATTR amyloidosis cohort study were the following: (1) a worsening of LV GLS values is correlated with an increase in NT-proBNP levels; (2) an increase in LA GLS is associated with MCP1 levels and reduced NT-proBNP; (3) an increase in IL6 and hsTnI plasma levels is correlated with poorer RV GLS values; (4) increased hsCRP values are associated with greater degrees of LV diastolic dysfunction (assessed through E/E′ ratio); and (5) the baseline klotho levels were significantly correlated with the 6MWT results.

Our average ATTR amyloidosis patient is an elderly (>75 years old) male with high blood pressure (69.2%) and a history of AF (46.2%) and LV hypertrophy, with two in four patients showing an extracardiac clinical sign and mild to moderate HF symptoms according to their NYHA class (>85% in NYHA I–II). According to laboratory data, median NT-proBNP levels were significantly high (when compared to the functional class of the majority of our cohort (NYHA I–II). This is also in line with the general knowledge that in CA, there is a great discordance between NT-ProBNP levels and HF clinical signs [12]. These results are similar to those from other studies depicting different cohorts of cardiac ATTR amyloidosis [13,14]. 

When facing diagnosis and prognosis in patients with CA, transthoracic echocardiogram has proved useful. Parameters regarding LV wall thickness and diastolic (Doppler E wave/e´ wave velocities, tricuspid regurgitation velocity, LA volume) and systolic function (LV GLS and systolic longitudinal strain apex to base ratio) have been established as non-invasive diagnostic criteria for CA. Even though they do not provide a full diagnosis alone, their importance lies in that they raise clinical suspicion and have a prognostic impact [12,15]. 

LV global longitudinal strain is an indicator of systolic function. It is expressed in negative values because it represents a shortening of the myocardium. In patients with CA, LV GLS is significantly reduced and its reduction (in absolute numbers) is associated with long-term prognosis [6]. It is also a great predictor of survival and response to therapy in AL amyloidosis [16]. Its association with elevated natriuretic peptide levels in chronic systolic HF and its relationship with increased risk of mortality and hospitalizations are well known [17]. High levels of NT-proBNP in patients with ATTR amyloidosis imply an increased risk of death and hospitalization. Levels of >3000 pg/mL are considered as an advanced-disease-stage parameter in different prognosis staging scores [18,19]. As we have already mentioned, in our cohort of ATTR amyloidosis patients, poorer values of LV GLS (closer to 0) were only associated with greater levels of NT-proBNP. This reinforces the fact that both parameters are strongly related with prognosis in this group.

On the other hand, the LA strain reservoir is a novel modality of LA function assessment. It has recently been inversely associated with the risk of HFpEF and BNP levels [20,21]. It also reflects the degree of atrial fibrosis and it is already altered long before the LA starts being dilated [22]. In our study, it inversely correlated with NT-proBNP levels, which is expected in the context of increased pressure in the intraventricular region and, subsequently, in the LA. Moreover, we found a clear correlation between LA strain and MCP1 levels. This biomarker has an essential role in different inflammatory processes, acting as a chemoattractant factor, gathering monocytes in the myocardium [23] and possibly increasing the degree of atrial fibrosis.

Other inflammatory markers, such as IL6, are also strongly associated with cardiac dysfunction. IL6 is an inflammatory marker with an important connection with coronary artery disease and the risk of HF [24,25]. IL6 exerts a negative inotropic effect on cardiac myocytes, worsening peak systolic circumferential strain values [26]. Our results are in line with this evidence, showing that elevated IL6 levels were associated with worse RV GLS values, which is the same as saying that elevated IL6 levels imply a greater degree of RV dysfunction. 

On the other hand, hsCRP has been independently linked with diastolic dysfunction in patients with HFpEF before [27] and with an overall poor long-term prognosis in HF patients [28]. Our results reinforce the former impression: patients with greater levels of hsCRP seemed to have greater degrees of diastolic dysfunction (assessed through the E/E′ ratio).

As we can see, there are some disparities in some of the correlations found, especially regarding inflammation and RV function: galectin 3 was only correlated with TAPSE, whereas IL6 only correlated with RV strain. RV strain identifies higher rates of RV dysfunction than classic TAPSE and fractional area changes [29]. This could suggest that IL6 is associated with earlier stages of RV dysfunction (during which RV strain is usually the only parameter affected), whereas galectin 3 would be related to more advanced stages when TAPSE is already altered.

Last but not least, mineral metabolism biomarkers seem to have a greater relationship with functional capacity. Klotho is an “ageing suppression” hormone. Its low levels have been associated with greater cognitive and functional decline and an increased likelihood of frailty and disability in elderly patients. In addition, its low plasma expression is related to low physical performance [30]. Our findings show that baseline klotho levels were significantly correlated with the 6MWT results; higher baseline klotho levels in patients with ATTR amyloidosis were associated with better functional capacity assessed through 6MWT. No other mineral metabolism biomarker was related with either cardiac function parameters or functional capacity. We must take into account that 23.1% of our patients had a certain degree of neurological dysfunction, which could have distorted the 6MWT results and the other possible associations. Nevertheless, and regardless of this point, klotho levels had a significant correlation with the patients’ performance in this functional capacity test.

As a result, these results could lead towards finding new methods of determining high-risk clinical profiles. It seems that higher inflammatory levels (IL6, MCP1) are associated with greater degrees of myocardial dysfunction (LA GLS, RV GLS, NT-ProBNP levels) and possibly worse prognosis. On the other hand, these correlations could suggest new therapeutic targets and therapies which could work adjunctive to the use of tafamidis (which prevents misfolding) [3] and, therefore, influence the clinical course of this disease. 

### Limitations

This study has the limitations inherent to an observational prospective single-center study. In addition, we must also consider the small number of patients included. Despite all this, significant correlations were found between biomarkers, cardiac function and clinical performance parameters.

In addition, it should be taken into account that the prevalence of alterations in the levels of these selected biomarkers in ATTR amyloidosis are currently unknown and therefore calculating the sample size according to them has not been possible. 

Also, the echocardiographic assessment was performed by a single operator. This could have introduced an inherent measurement bias within our data.

It is indeed important to take into account that the results regarding the 6MWT could be influenced by the presence of neurological dysfunction (peripheral polyneuropathy) in patients with ATTR amyloidosis (23.1% of our patients), so the conclusions regarding this test should be taken with caution.

## 5. Conclusions

In a cohort of patients with ATTR amyloidosis, a significant correlation was found between inflammatory biomarkers and cardiac dysfunction assessed through LV and RV GLS and LA reservoir strain. On the other hand, klotho baseline levels were associated with physical performance. 

More studies are needed in order to clarify the possible role of these inflammatory and mineral metabolism biomarkers in the prognosis of patients with ATTR amyloidosis and whether they can be considered as potential therapeutic targets that could modify the natural course of this disease.

## Figures and Tables

**Table 1:** Baseline characteristics of total population.

Patient Description	N = 13
Age, (years)	79 (76–85.5)
Women, *n* (%)	6 (46.2)
Diabetes, *n* (%)	4 (30.8)
Smokers, *n* (%)	3 (23.1)
Dyslipidemia, *n* (%)	10 (76.9)
Arterial hypertension, *n* (%)	9 (69.2)
Obesity, *n* (%)	3 (23.1)
Atrial fibrillation, *n* (%)	6 (46.2)
Ischemic heart disease, *n* (%)	1 (7.7)
Previous revascularization, *n* (%)	1 (7.7)
Pacemaker carriers, *n* (%)	0 (0)
Previous hospitalization for HF, *n* (%)	4 (30.8)
Previous stroke, *n* (%)	1 (7.7)
Chronic lung disease, *n* (%)	2 (15.4)
NYHA class, *n* (%)	
I	4 (30.8)
II	8 (61.5)
III	1 (7.7)
Sinus rhythm, *n* (%)	9 (69.2)
LBBB, *n* (%)	0 (0)
RBBB, *n* (%)	1 (7.7)
Low voltage, *n* (%)	3 (23.1)
Medical treatment	
Anticoagulation, *n* (%)	5 (38.5)
Apixaban, *n* (%)	2 (40.0)
Edoxaban, *n* (%)	2 (40.0)
Acenocumarol, *n* (%)	1 (20.0)
Antiplatelets (aspirin), *n* (%)	1 (7.7)
ACEIs, *n* (%)	3 (23.1)
ARBs, *n* (%)	4 (30.8)
ARNI, *n* (%)	0 (0)
Beta-blockers, *n* (%)	6 (46.2)
Mineralocorticoid receptor antagonists, *n* (%)	2 (15.4)
SGLT2i, *n* (%)	1 (7.7)
Loop diuretics, *n* (%)	8 (61.5)
Digoxin, *n* (%)	0 (0)
Antiarrhythmic drugs, *n* (%)	1 (7.7)
Clinical signs	
Carpal tunnel syndrome, *n* (%)	3 (23.1)
Lumbar spinal stenosis, *n* (%)	4 (30.8)
Bicep tendon rupture, *n* (%)	3 (23.1)
Polyneuropathy, *n* (%)	3 (23.1)
Dementia, *n* (%)	1 (7.7)
Laboratory values	
Creatinine, (mg/dL)	1.0 (1.0–1.5)
Estimated glomerular filtration rate, (mL/Min)	61 (48.5–83.0)
Hemoglobin, (g/dL)	14 (13.0–14.5)
Leucocytes, (*n*/mm^3^)	6000 (4500–8000)
NT-ProBNP, (ng/mL)	1340 (867–1598)
Proteins (serum), (g/dL)	7 (6.8–7)
Proteins (urine), (g/dL)	4.0 (4.0–23.5)
Albumin (serum), (g/dL)	4.0
Microalbuminuria	27 (7.5–89.5)
Echocardiographic parameters	
LVEF, (%)	52.0 (46.1-54.5)
LVEF < 40%, *n* (%)	0 (0)
LVTDD, (mm)	39.0 (32.0-43.5)
IVS thickness, (mm)	17 (15.0-18.5)
LV hypertrophy, *n* (%)	13 (100)
E/E′	14.1 (12.8–18.0)
Mitral regurgitation (grade II/III), *n* (%)	1 (7.7)
Aortic stenosis (any degree), *n* (%)	1 (7.7)
RVTDD, (mm)	43.0 (34.5–46.0)
PASP, (mmHg)	43.0 (37.0–53.0)
Aortic stenosis, *n*	1
Mean Grad (mmHg)	19.5
Max V (m/s)	3.1
Genetic testing	(n = 13)
Positive (ATTR v), *n* (%)	2 (15.4)
Negative (ATTR wt), *n* (%)	11 (84.6)

Data are presented as medians (interquartile ranges) or percentages. ACEIs, angiotensin-converting-enzyme inhibitors; ARBs, angiotensin II receptor blockers; ARNI, angiotensin receptor and neprilysin inhibitors; HF, heart failure; IVS, interventricular septum; LBBB, left bundle branch block; LVEF, left ventricular ejection fraction; LVEF, left ventricular ejection fraction; LVTDD, left ventricular telediastolic diameter; LVOT, left ventricular outflow tract; PASP, pulmonary artery systolic pressure; RBBB, right bundle branch block; RVTDD, right ventricular telediastolic diameter; SGLT2i, sodium-glucose cotransporter 2 inhibitors.

**Table 2:** Strain data, biomarker levels and 6MWT for the selected 13 patients.

Echocardiographic Parameters	N = 13
LVEF, (%)	52.0 (46.1–54.5)
LV mass, (g)	225.9 (169.6–343.5)
E/E′ TAPSE, (mm)	14.1 (12.8–18.0) 18 (16–24)
LV GLS, (%)	−10 (−15–−6)
RV 4CLS, (%)	−10.3 (−11.5–−6.1)
LA strain, (%)	9.2 (3.5–11.0)
Biomarker levels	
hsTnI, (pg/mL)	127.6 (83.5–152.1)
CKMb, (ng/mL)	1.79 (1.4–2.5)
NT-ProANP, (pg/mL)	68.9 (62.8–75.9)
NT-ProBNP, (ng/mL)	1340 (867–1598)
Il 6, (pg/mL)	2.7 (0–7.1)
TIM 1, (pg/mL)	115.0 (0–181.5)
Galectin 3, (pg/mL)	7.4 (5.1–8.5)
hsCRP, (mg/dL)	1.59 (0.8–2.3)
MCP1, (pg/mL)	167.0 (133.5–189.0)
PTH, (ng/L)	92.7 (55.5–132.5)
Klotho, (pg/mL)	552.0 (465.0–698.3)
FGF23, (RU/mL)	214.5 (105.5–304.3)
Calcidiol, (ng/mL)	32.8 (18.1–39.8)
6 min walk test (m)	280 (230–310)

Data are presented as medians (interquartile ranges) or percentages. CKMb, creatinine kinase myocardial band; FGF23, fibroblast growth factor 23; GLS, global longitudinal strain; hsCPR, high sensitivity C reactive protein; hsTnI, high-sensitivity troponin I; IL6, interleukin 6;LA, left atrium; LV, left ventricle; LVEF, left ventricular ejection fraction; MCP1, monocyte chemoattractant protein 1; PTH, parathormone; RV4CLS, right ventricle 4 chambers longitudinal strain; TAPSE, tricuspid annular plane systolic excursion; TIM 1, T cell immunoglobulin and mucin domain 1.

**Table 3:** Correlations between serum biomarkers and different cardiac variables.

		ProBNP	IL6 *	Galectin 3	hsCPR *	MCP1	hsTnI	Klotho
6MWT	R	−0.135	−0.185	−0.165	−0.246	−0.241	−0.09	0.567
	P	0.660	0.546	0.628	0.422	0.427	0.978	0.044
LVEF	R	−0.467	−0.071	0.547	0.236	0.170	0.334	0.050
	P	0.108	0.867	0.081	0.460	0.578	0.265	0.872
TAPSE	R	−0.653	−0.105	0.759	0.299	0.204	0.545	−0.082
	P	0.016	0.804	0.007	0.344	0.504	0.054	0.791
E/E′ ratio	R	0.352	0.286	0.340	0.576	0.029	0.130	0.085
	P	0.239	0.493	0.307	0.050	0.924	0.673	0.783
RV GLS *	R	0.029	0.881.	−0.463	−0.084	0.464	0.616	0.247
	P	0.925	0.004	0.151	0.796	0.110	0.025	0.415
LA GLS	R	−0.727	0.376	0.530	0.275	0.553	−0.522	−0.320
	P	0.005	0.359	0.094	0.386	0.050	0.067	0.286
LV GLS	R	0.610	0.138	−0.360	−0.089	−0.371	0.207	−0.069
	P	0.027	0.745	0.277	0.784	0.211	0.497	0.822

6MWT: six-minute walk test; GLS, global longitudinal strain; hsCPR, high-sensitivity C reactive protein; IL6, interleukin 6; LA, left atrium; LV, left ventricle; LVEF, left ventricular ejection fraction; MCP1, monocyte chemoattractant protein 1; RV, right ventricle; TAPSE, tricuspid annular plane systolic excursion. * Variables with non-normal distribution. Spearman test was used.

## Data Availability

The data presented in this study are available on request from the corresponding author due to privacy and ethical reasons.

## References

[1] AbouEzzeddine O.F., Davies D.R., Scott C.G., Fayyaz A.U., Askew J.W., McKie P.M., Noseworthy P.A., Johnson G.B., Dunlay S.M., Borlaug B.A. (2021). Prevalence of Transthyretin Amyloid Cardiomyopathy in Heart Failure with Preserved Ejection Fraction. JAMA Cardiol..

[2] González-López E., Gallego-Delgado M., Guzzo-Merello G., de Haro-Del Moral F.J., Cobo-Marcos M., Robles C., Bornstein B., Salas C., Lara-Pezzi E., Alonso-Pulpon L. (2015). Wild-type transthyretin amyloidosis as a cause of heart failure with preserved ejection fraction. Eur. Heart J..

[3] Ruberg F.L., Maurer M.S. (2024). Cardiac Amyloidosis Due to Transthyretin Protein: A Review. JAMA.

[4] Koike H., Okumura T., Murohara T., Katsuno M. (2021). Multidisciplinary Approaches for Transthyretin Amyloidosis. Cardiol. Ther..

[5] Jaiswal V., Agrawal V., Khulbe Y., Hanif M., Huang H., Hameed M., Shrestha A.B., Perone F., Parikh C., Gomez S.I. (2023). Cardiac amyloidosis and aortic stenosis: A state-of-the-art review. Eur. Heart J. Open.

[6] Chacko L., Martone R., Bandera F., Lane T., Martinez-Naharro A., Boldrini M., Rezk T., Whelan C., Quarta C., Rowczenio D. (2020). Echocardiographic phenotype and prognosis in transthyretin cardiac amyloidosis. Eur. Heart J..

[7] Gillmore J.D., Maurer M.S., Falk R.H., Merlini G., Damy T., Dispenzieri A., Wechalekar A.D., Berk J.L., Quarta C.C., Grogan M. (2016). Nonbiopsy Diagnosis of Cardiac Transthyretin Amyloidosis. Circulation.

[8] Luciani M., Troncone L., Monte F.D. (2018). Current and future circulating biomarkers for cardiac amyloidosis. Acta Pharmacol. Sin..

[9] Coelho T., Maia L.F., Martins da Silva A., Waddington Cruz M., Planté-Bordeneuve V., Lozeron P., Suhr O.B., Campistol J.M., Conceição I.M., Schmidt H.H. (2012). Tafamidis for transthyretin familial amyloid polyneuropathy: A randomized, controlled trial. Neurology.

[10] Maurer M.S., Schwartz J.H., Gundapaneni B., Elliott P.M., Merlini G., Waddington-Cruz M., Kristen A.V., Grogan M., Witteles R., Damy T. (2018). Tafamidis Treatment for Patients with Transthyretin Amyloid Cardiomyopathy. N. Engl. J. Med..

[11] Berk J.L., Suhr O.B., Obici L., Sekijima Y., Zeldenrust S.R., Yamashita T., Heneghan M.A., Gorevic P.D., Litchy W.J., Wiesman J.F. (2013). Repurposing diflunisal for familial amyloid polyneuropathy: A randomized clinical trial. JAMA.

[12] Garcia-Pavia P., Rapezzi C., Adler Y., Arad M., Basso C., Brucato A., Burazor I., Caforio A.L., Damy T., Eriksson U. (2021). Diagnosis and treatment of cardiac amyloidosis: A position statement of the ESC Working Group on Myocardial and Pericardial Diseases. Eur. Heart J..

[13] Connors L.H., Sam F., Skinner M., Salinaro F., Sun F., Ruberg F.L., Berk J.L., Seldin D.C. (2016). Heart Failure Resulting from Age-Related Cardiac Amyloid Disease Associated with Wild-Type Transthyretin: A Prospective, Observational Cohort Study. Circulation.

[14] Pinney J.H., Whelan C.J., Petrie A., Dungu J., Banypersad S.M., Sattianayagam P., Wechalekar A., Gibbs S.D., Venner C.P., Wassef N. (2013). Senile Systemic Amyloidosis: Clinical Features at Presentation and Outcome. J. Am. Heart Assoc..

[15] Kittleson M.M., Maurer M.S., Ambardekar A.V., Bullock-Palmer R.P., Chang P.P., Eisen H.J., Nair A.P., Nativi-Nicolau J., Ruberg F.L., American Heart Association Heart Failure and Transplantation Committee of the Council on Clinical Cardiology (2020). Cardiac Amyloidosis: Evolving Diagnosis and Management: A Scientific Statement from the American Heart Association. Circulation.

[16] Cohen O.C., Ismael A., Pawarova B., Manwani R., Ravichandran S., Law S., Foard D., Petrie A., Ward S., Douglas B. (2022). Longitudinal strain is an independent predictor of survival and response to therapy in patients with systemic AL amyloidosis. Eur. Heart J..

[17] Gaborit F., Bosselmann H.S., Tønder N., Iversen K., Kümler T., Kistorp C., Sölétormos G., Goetze J.P., Schou M. (2015). Association between left ventricular global longitudinal strain and natriuretic peptides in outpatients with chronic systolic heart failure. BMC Cardiovasc. Disord..

[18] Grogan M., Scott C.G., Kyle R.A., Zeldenrust S.R., Gertz M.A., Lin G., Klarich K.W., Miller W.L., Maleszewski J.J., Dispenzieri A. (2016). Natural History of Wild-Type Transthyretin Cardiac Amyloidosis and Risk Stratification Using a Novel Staging System. J. Am. Coll. Cardiol..

[19] Gillmore J.D., Damy T., Fontana M., Hutchinson M., Lachmann H.J., Martinez-Naharro A., Quarta C.C., Rezk T., Whelan C.J., Gonzalez-Lopez E. (2018). A new staging system for cardiac transthyretin amyloidosis. Eur. Heart J..

[20] Aung S.M., Güler A., Güler Y., Huraibat A., Karabay C.Y., Akdemir I. (2017). Left atrial strain in heart failure with preserved ejection fraction. Herz.

[21] Boe E., Smiseth O.A. (2022). Left atrial strain imaging: Ready for clinical implementation in heart failure with preserved ejection fraction. Eur. Heart J. Cardiovasc. Imaging.

[22] Saraiva R.M., Demirkol S., Buakhamsri A., Greenberg N., Popović Z.B., Thomas J.D., Klein A.L. (2010). Left atrial strain measured by two-dimensional speckle tracking represents a new tool to evaluate left atrial function. J. Am. Soc. Echocardiogr. Off. Publ. Am. Soc. Echocardiogr..

[23] Blanco-Colio L.M., Méndez-Barbero N., Pello Lázaro A.M., Aceña Á., Tarín N., Cristóbal C., Martínez-Milla J., González-Lorenzo Ó., Martín-Ventura J.L., Huelmos A. (2021). MCP-1 Predicts Recurrent Cardiovascular Events in Patients with Persistent Inflammation. J. Clin. Med..

[24] IL6R Genetics Consortium Emerging Risk Factors Collaboration (2012). Interleukin-6 receptor pathways in coronary heart disease: A collaborative meta-analysis of 82 studies. Lancet Lond. Engl..

[25] Plenz G., Song Z.F., Tjan T.D., Koenig C., Baba H.A., Erren M., Flesch M., Wichter T., Scheld H.H., Deng M.C. (2001). Activation of the cardiac interleukin-6 system in advanced heart failure. Eur. J. Heart Fail..

[26] Yan A.T., Yan R.T., Cushman M., Redheuil A., Tracy R.P., Arnett D.K., Rosen B.D., McClelland R.L., Bluemke D.A., Lima J.A. (2010). Relationship of interleukin-6 with regional and global left-ventricular function in asymptomatic individuals without clinical cardiovascular disease: Insights from the Multi-Ethnic Study of Atherosclerosis. Eur. Heart J..

[27] Michowitz Y., Arbel Y., Wexler D., Sheps D., Rogowski O., Shapira I., Berliner S., Keren G., George J., Roth A. (2008). Predictive value of high sensitivity CRP in patients with diastolic heart failure. Int. J. Cardiol..

[28] Tang W.H.W., Shrestha K., Van Lente F., Troughton R.W., Martin M.G., Borowski A.G., Jasper S., Klein A.L. (2008). Usefulness of C-Reactive Protein and Left Ventricular Diastolic Performance for Prognosis in Patients with Left Ventricular Systolic Heart Failure. Am. J. Cardiol..

[29] Prihadi E.A., van der Bijl P., Dietz M., Abou R., Vollema E.M., Marsan N.A., Delgado V., Bax J.J. (2019). Prognostic Implications of Right Ventricular Free Wall Longitudinal Strain in Patients with Significant Functional Tricuspid Regurgitation. Circ. Cardiovasc. Imaging.

[30] Valenzuela P.L., Cobo F., Diez-Vega I., Sánchez-Hernández R., Pedrero-Chamizo R., Verde-Rello Z., González-Gross M., Santiago C., Ruiz M.P. (2019). Physical performance, plasma S-klotho, and all-cause mortality in elderly dialysis patients: A prospective cohort study. Exp. Gerontol..

